# Development of 10 L mass culture system of human induced pluripotent stem cells with intermittent agitation using plastic fluid

**DOI:** 10.3389/fbioe.2025.1664723

**Published:** 2025-11-18

**Authors:** Tomohiro Tokura, Riku Yamamoto, Masahiro Kino-oka

**Affiliations:** 1 Department of Biotechnology, Graduate School of Engineering, The University of Osaka, Osaka, Japan; 2 ZACROS Corporation, Hyogo, Japan; 3 Research Base for Cell Manufacturability, Graduate School of Engineering, The University of Osaka, Osaka, Japan

**Keywords:** human induced pluripotent stem cells, process design, closed culture system, aggregate culture, plastic fluid

## Abstract

Human induced pluripotent stem cells (hiPSCs) are crucial for cell therapy and regenerative medicine. The development of high-yield and stable mass-culture technologies is essential for the industrialization of hiPSCs. In this study, we proposed a design procedure for the scale-up of hiPSCs and evaluated a mass culture system at a 10 L scale, which is currently challenging. We developed a design procedure for a hiPSC aggregate culture system based on cell manufacturability. Considering the biological aspects, including not only cell behavior but also aggregate behavior, the input variables of the engineering aspects were identified. To mitigate the hydrodynamic force of the fluid flow caused by agitation, we proposed intermittent agitation using a plastic fluid. This method maintained the oxygen supply and aggregate dispersion with minimal agitation using fluid plasticity. Moreover, designing mass cultures requires the establishment of aseptic processing. We developed a single-use bioreactor and closed system, along with a medium exchange and preparation process to ensure aseptic processing. After designing the mass culture system, small-scale model experiments were carried out using a 1 L bioreactor. In three independent trials, the specific growth rate of hiPSCs was found to be similar to that of the conventional small stirred bioreactor. In addition to the above-mentioned culture design, the addition of a Rho-associated coiled-coil containing protein kinase (ROCK) inhibitor was required to maintain the aggregate structure at the 10 L scale. We stably performed 10 L cultures three times, and the specific growth rate was comparable to that on the 1 L scale. The final cell number of hiPSCs reached (1.09 ± 0.02) × 10^10^ cells. These findings provide a procedure for scaling up the design and contribute to the development of mass culture systems that ensure reproducible and stable hiPSC cultures.

## 1 Introduction

For the industrialization of cell-based therapies, hiPSCs are powerful tools for application in regenerative therapy. Immunological rejection can be suppressed using HLA haplotype-homozygous hiPSCs (de Rham and Villard, 2014); therefore, allogeneic transplantation of tissues or cells derived from them has the potential to be applied to a wide range of patients. Recent clinical trials have demonstrated the therapeutic potential for allogenic hiPSC-derived cells for various diseases (Sugai et al., 2021; Miyagawa et al., 2022; Akiba et al., 2023; Sawamoto et al., 2025) confirming their safety and efficacy. To provide these therapies affordably and widely, the establishment of a large-scale culture system is critical for cell manufacturing. The concept of “cell manufacturability” has been proposed in the process design of cell manufacturing (Kino-oka et al., 2019). This concept is defined as “the attainment of the desired capability of a cell manufacturing process by bridging the gap between its biological and engineering aspects.” In accordance with this concept, understanding the characteristics of cells and designing their processes are crucial for the development of large-scale culture systems for hiPSCs.

Understanding cell and aggregate behaviors is crucial for hiPSC culture design. hiPSCs are anchorage-dependent and undergo apoptosis without attachment (Ohgushi et al., 2010). In suspension cultures without scaffolds, hiPSCs connect with other cells and form aggregates. Subsequently, these cells divide and the aggregate size increases. During passage, dispersed single cells are inoculated into the suspension system to form aggregates. However, the number of surviving cells 24 h after seeding decreases owing to apoptosis (Kato et al., 2018). In addition, the initial aggregate diameter affects proliferation potential (Nath et al., 2017a). Therefore, suitable management of aggregate formation is required as a first step in the cell culture process. As the next step in cell proliferation, maintenance of the aggregate structure, which means the prevention of aggregate collapse and coalescence, is required. During this step, hiPSCs secrete extracellular matrix (ECM) to sustain their aggregate structure. Insufficient ECM production leads to the collapse of aggregates and cell death (Kim et al., 2018). In contrast, fibrillated collagen type I secreted from hiPSCs forms a shell-like structure around the periphery of the aggregate in the late stages of culture, which suppresses proliferation (Nath et al., 2017a). Hence, techniques for controlling the ECM production and structure are necessary. Additionally, when multiple aggregates coexist within the environment, hiPSC aggregates can agglomerate through E-cadherin-mediated cell-cell interactions (Stephen M et al., 2004). The increase in aggregate diameter owing to excessive coalescence induces contact inhibition and diffusive limitation inside the aggregate, which suppresses cell proliferation (Wu et al., 2014; Nath et al., 2017a; Nakamura, 2024). Therefore, the design of two independent processes, the formation of many homogeneously sized aggregates without losing cell number (first step), and the expansion of cell aggregates while maintaining the aggregate structure (second step), is needed based on biological behaviors.

Current culture systems typically combine aggregate formation and expansion in a single step. While small-scale dimple plates enable controlled formation of <10^7^ homogeneously sized aggregates (Karp et al., 2007; Limraksasin et al., 2020; Suenaga et al., 2022), dynamic suspension systems offer scale-up potential by using agitation to prevent sedimentation, promote cell aggregation, and enhance oxygen supply. However, these systems face challenges in controlling aggregate size and preventing coalescence during scale-up. Conversely, hydrodynamic forces can damage cells. Shear stress is the main cause of damage to animal cells (Papoutsakis, 1991; Elias et al., 1995; Mollet et al., 2007; Hu et al., 2011), and hiPSCs are particularly sensitive to this stress (Ismadi et al., 2014). Additionally, the normal stress, which is applied in the vertical direction to the cell aggregate surface, must be considered. This stress is also developed by the hydrodynamic force or collision with other aggregates and can lead to their collapse (Kim et al., 2018). Plastic fluids have been employed in hiPSC suspension culture systems to prevent coalescence and collapse of aggregates (Otsuji et al., 2014; Yamamoto and Kino-oka, 2021). While intermittent agitation strategies have been explored to guide cellular differentiation (Correia et al., 2014; Ting et al., 2014), their application for large-scale expansion in conventional fluids is limited by aggregate sedimentation during static phases. The use of plastic fluids offers a potential solution to this challenge. This fluid has microstructures of polymer in the liquid that exhibit a solid-like phase at stresses below the yield stress; when a force greater than the yield stress is applied, the microstructures break down and the fluid flows. Additionally, this fluid can be agitated during culture while maintaining its properties (Horiguchi et al., 2021). Complete control of fluid flow inside bioreactors is almost impossible; therefore, it is necessary to define the role of agitation depending on the processes of aggregate formation and cell expansion.

Additionally, hiPSCs typically exhibit high lactate production capacity but have low tolerance to the accumulation (Nath et al., 2017b; Horiguchi et al., 2018). This indicates that hiPSC culture requires medium exchange not only for substrate feeding but also for waste removal. Medium exchange in suspension culture requires separating cells from spent medium through gravity sedimentation, centrifugation, or membrane separation. While gravity separation is simple and cost-effective, it poses scale-up limitations due to coalescence risks during extended sedimentation times. Continuous centrifugation, which is widely used in monoclonal antibody production, can easily be scaled up in closed systems (Jungbauer, 2013). However, sedimentation owing to centrifugal forces leads to the formation of aggregate clumps and increases the risk of coalescence. Membrane separation, especially crossflow filtration, such as tangential flow filtration (TFF) or alternating tangential flow filtration (ATF), is commonly used for single-cell suspension (Clincke et al., 2013; Kelly et al., 2014; Walther et al., 2019). Membrane separation methods mainly employ hollow fibers, which provide a large surface area in closed systems. The challenges of TFF are the pressure drop and decrease in separation efficiency due to membrane fouling (Jungbauer, 2013; Kelly et al., 2014). Since hiPSCs tend to aggregate easily, medium exchange operations become more difficult, and a specific design targeting cell aggregates is required.

These hiPSC culture processes must be performed aseptically. While aseptic processing is usually performed inside a safety cabinet or closed isolator system in dish-scale cultures, the construction of a closed culture system is essential for large-scale bioreactor operations. A closed system for each apparatus and aseptic tubing connection should be equipped for liquid handling. Furthermore, the ancillary materials used for these processes must be prepared aseptically. The culture medium for hiPSCs contains high molecular weight proteins such as growth factors; therefore, a small volume of supplements ranging from 1/10 to 1/1000 should be mixed into the basal medium. These supplements have a short shelf life owing to their low thermal stability, and medium preparation must be performed aseptically at the time of use.

In this study, we aimed to expand large number of hiPSCs in a 10 L suspension culture system based on cell manufacturability. A scale-up design procedure was proposed, and the required unit processes were identified as the aggregate formation process, cell expansion process, medium exchange process, and a sub-process of medium preparation, all within a closed system. The developed 10 L culture system achieved 1.09 × 10^10^ cells while maintaining an undifferentiated state and trilineage differentiation potential.

## 2 Materials and methods

### 2.1 Design methodology for hiPSC mass culture system

**FIGURE 1:**
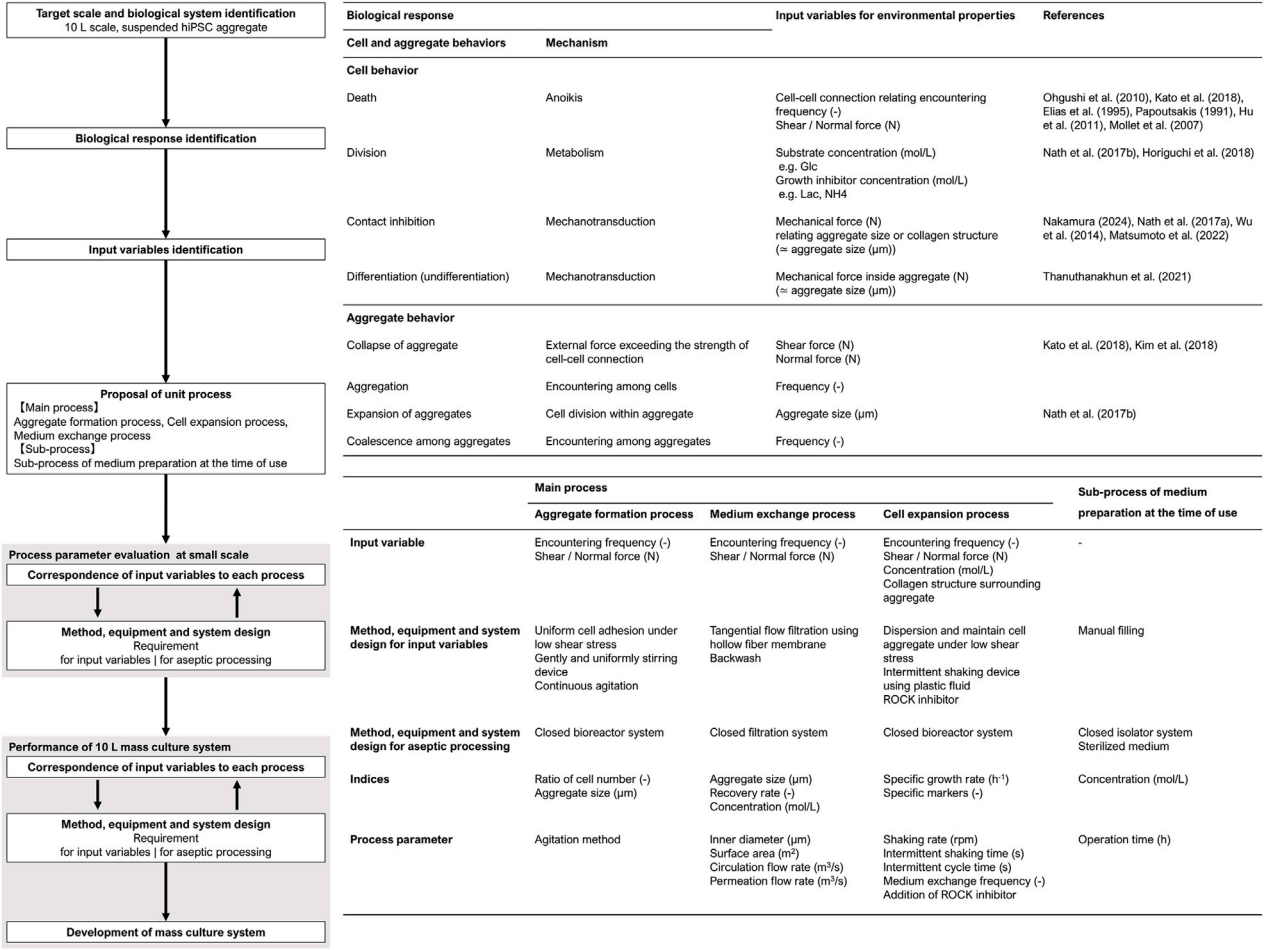
Flowchart of scale-up design for hiPSCs aggregate culture system.

This study proposed a scale-up design methodology for large-scale hiPSC culture processes based on cell manufacturability. Based on this concept, the cell manufacturing process was designed, as shown in the flowchart in Figure 1. The first step in the design procedure is the identification of the target scale and biological system. In this study, the target volume was set to 10 L, and hiPSCs were grown as suspended aggregates. According to this system, biological responses were categorized into cellular-level and aggregate-level behaviors. Cellular behavior included death, division, differentiation, and contact inhibition, whereas aggregate behavior included the aggregation, expansion, coalescence, and collapse. These behaviors are controlled by input variables that regulate environmental properties. Environmental properties refer to the components of the culture system, which consists of a solid phase (scaffold and neighboring cells), a liquid phase (materials, heat, force, and motion), and a gas phase. Since these properties influence the culture system throughout the culture process, it is necessary to divide the total mass culture system into unit processes. This approach allows the identification of requirements in each unit process while ensuring their integration into the total mass culture system.

The requirements of this system were as follows: (1) formation of homogeneous and suitably sized cell aggregates, (2) maintenance of suitable medium component concentrations, and (3) maintenance of aggregate structures without aggregate decay and coalescence. Therefore, we designed the following three-unit process: (1) aggregate formation, (2) medium exchange, and (3) cell expansion. In addition to these biological and engineering considerations, all cell manufacturing processes must be performed aseptically. Therefore, closed systems were designed for each unit process, and a supporting sub-process for medium preparation was also developed (4).

After determining the input variables for each unit process, the process must be designed to satisfy both the corresponding input variables and aseptic processing requirements. The configuration of methods, equipment, and systems requires interactive refinement and adjustment of the process parameters through repeated small-scale evaluations. Once an acceptable system was established, the design proceeded to large-scale adaptation. If certain parameters were found to be inapplicable at a large scale, minimal additional condition adjustments were required. The integrated mass culture system was evaluated after the configuration of each unit process was completed. The performance of this culture system was assessed in terms of the cell number, yield, maintenance of the undifferentiated state, and differentiation potential.

#### 2.1.1 Aggregate formation process

The objective of the aggregate formation process was to prepare homogeneous aggregates without losing the cells. Previous reports have shown that homogeneous aggregates prepared under agitation conditions exhibit a coefficient of variation of approximately 0.2–0.3 (Miranda et al., 2015; 2016), and the initial hiPSC aggregate diameters of 100–220 µm have good potential for their growth (Abbasalizadeh et al., 2012; Nath et al., 2017a). Under suspension conditions, the cells require attachment to prevent apoptosis and are easily damaged, causing necrosis by shear stress.

Since the liquid flow regulates these phenomena, we selected two agitation systems: orbital shaking and stirring with a delta paddle impeller. These agitation systems form homogeneous aggregates in low shear-stress environments (Matsuura et al., 2012; Horiguchi et al., 2023). These two agitation systems were compared in terms of the identified indices, aggregate diameter, and the distribution and ratio of cell numbers after 48 h of preparation.

#### 2.1.2 Medium exchange process

The objective of the medium-exchange process was to refresh the medium components on a large scale without causing cell loss. hiPSC proliferation is highly sensitive to lactic acid accumulation and its concentration should be managed below 1.0 g/L (Nath et al., 2017b). In addition, due to the complexity of this process, a higher exchange efficiency is important. Since we used plastic fluid for the expansion process, the cell aggregates could neither be settled by gravity nor be efficiently sedimented without excessive centrifugal force. Therefore, hollow fiber membrane filtration was used to separate aggregates from the culture medium. In this filtration system, cell trapping decreases the recovery rate (Wang et al., 2017). A concentration operation using narrow hollow fibers increases the encountering frequency and risk of clogging. In addition, the higher linear velocity inside the narrow hollow fibers may damage the cells through shear and normal stresses. Thus, the prevention of membrane fouling is essential for the large-scale separation of cell aggregates from the medium.

**FIGURE 2:**
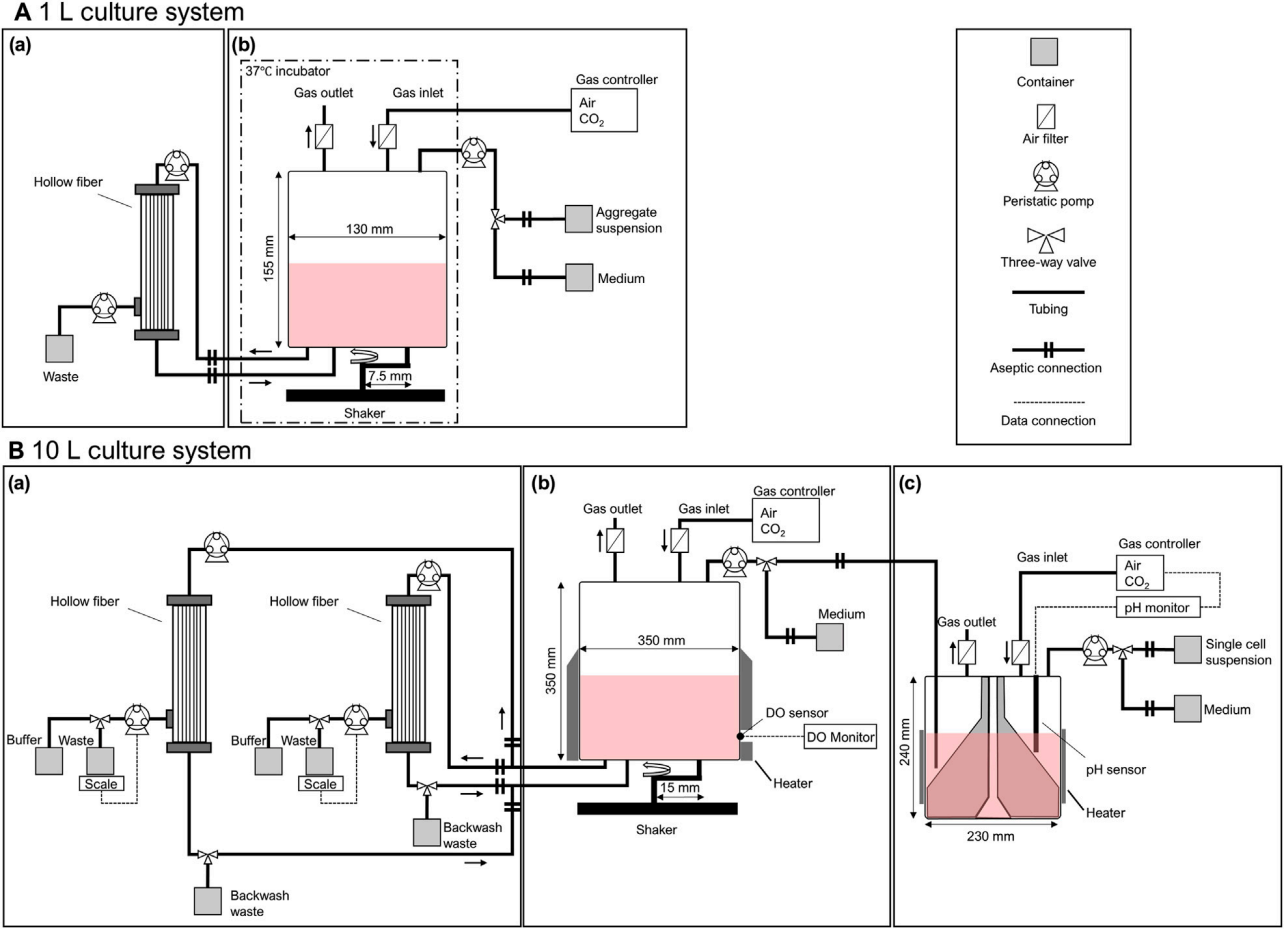
Schematic diagram of **(A)** 1 L and **(B)** 10 L mass culture systems for hiPSCs. The mass culture system consisted of **(a)** a medium exchange process with a tangential flow filtration system using hollow fibers, **(b)** a cell expansion process with intermittent agitation using a plastic fluid, and **(c)** an aggregate formation process with a delta paddle stirring device. Each process was aseptically conducted and maintained in a closed environment.

In this study, the balance between the circulation and permeation flows was investigated to prevent membrane fouling. Although lower permeation flow rates contribute to reduced membrane fouling, they also extend operation time. Therefore, the permeation flow rate was set at a level that prevented entrapment while implementing parallel operations to maintain efficiency (Figure 2Ba). The inner diameter of the hollow fibers was also investigated to prevent membrane clogging. Additionally, backwash operations were performed to mitigate fouling. This process was evaluated using medium concentration and recovery rate as indices of medium exchange.

#### 2.1.3 Cell expansion process

The objective of the cell expansion process was to achieve hiPSC proliferation in a manner comparable to conventional suspension culture systems. To maintain the proliferative capacity, it is essential to ensure an adequate oxygen supply while preserving aggregate structure. This requirement is satisfied by continuous agitation on a small scale; however, the hydrodynamic stress may exceed the range of aggregate tolerance when scaling up.

In this study, an intermittent agitation culture system using plastic fluid, which can suspend aggregates without agitation, was developed for a lower hydrodynamic stress environment. Oxygen was supplied by temporal short-term agitation, and the specific growth rate was evaluated to compare the performance of the culture system. Additionally, to improve the robustness of the aggregate structure for large-scale culture, we added a ROCK inhibitor. Exposure to Rho-associated coiled-coil containing protein kinase (ROCK) inhibitor (Y-27632; Wako Pure Chemical Industries, Japan) can promote the secretion of type I collagen (Kim et al., 2018) and maintain the aggregate structure (Matsumoto et al., 2022).

#### 2.1.4 Sub-process of medium preparation at the time of use

The objective of this sub-process was to aseptically prepare 10 L of fresh medium in containers at the time of use, which can be connected to the main processes in the closed system. For this process, we selected a commercially available medium (StemFit AK02N; Ajinomoto Co. Inc., Japan) used in plastic fluid cultures (Horiguchi et al., 2021; Yamamoto and Kino-oka, 2021). This medium has a short shelf life owing to the low thermal stability of some components and should be prepared at the time of use. Multiple medium bottles of various sizes were batched in large containers. In particular, the volume of supplements is very small, and loss during operation critically affects the medium component concentration. Each material is packaged in a different format and a series of operations must be performed through aseptic processing.

This study established an operating procedure for mixing twenty bottles of basal medium and a small amount of supplements to enable preparation at the time of use. The volume of the medium was precisely measured using an autopipette and subsequently mixed. An isolator system was used to perform these operations through aseptic processing. Due to the large number of materials, they must be installed in an aseptic environment multiple times. To facilitate rapid material transfer, a double-pass box isolator system (Supplementary Figure S1A) was used. This system enabled a two-step disinfection process similar to that used in cleanroom environments. Due to the parallel operation during medium exchange, two containers of 5 L size were prepared at the time of use.

### 2.2 Cell and culture conditions

The hiPSC line, 1383D2, was provided by the Center for iPS Research and Application (Kyoto University, Japan). Cells were routinely maintained on polystyrene substrates coated with a recombinant laminin-511 E8 fragment (iMatrix-511; Nippi Inc., Japan) in StemFit AK02N medium. For subculture, single cells were suspended with 10 µM Y-27632, subsequently inoculated at 2.5 × 10^3^ cells/cm^2^. Cells were incubated at 37 °C in a humidified atmosphere with 5% CO_2_, and medium exchange was performed after 24 h and every 2 days. On day 4, when cells reached an 80%–90% confluence, hiPSCs were treated with 5 mM ethylenediaminetetraacetic acid (EDTA) (0.5 mmol/L EDTA/PBS solution; Nacalai Tesque, Inc., Japan) and phosphate-buffered saline (PBS) with 10 µM ROCK inhibitor for 10 min at room temperature. The dissociation reagent (TrypLE Select^®^, Thermo Fisher Scientific Inc., USA) with 10 µM ROCK inhibitor was applied for 7 min at room temperature, and hiPSCs were dissociated into single cells by pipetting. After centrifugation, single hiPSCs were resuspended in fresh medium with 10 µM ROCK inhibitor. Viable cells were counted, and the cells were re-seeded onto new culture vessels.

### 2.3 Preparation process of hiPSC aggregates

Aggregate formation was performed in a 100 mL Erlenmeyer flask (AGC Techno Glass Co., Ltd., Japan), a 100 mL delta paddle stirring device (BWV-S10A; Able Co., Japan), and a 5 L delta paddle stirring device (prototype; Able Co., Japan). First, hiPSCs were cultured on culture dishes, then dissociated into single cells by 5 mM EDTA/PBS with 10 µM ROCK inhibitor. After centrifugation, single hiPSCs were re-suspended in a fresh medium. hiPSCs were inoculated at 1.0 × 10^8^ cells/L followed by 48 h of culture to form cell aggregates. 100 mL Erlenmeyer flask and 1L bioreactor were placed at an orbital shaker at 100 rpm and incubated at 37 °C in a humidified atmosphere with 5% CO_2_. The 100 mL and 5 L delta paddle stirring devices were placed on a magnetic stirrer, and the agitation rates were maintained at 40 and 20 rpm, respectively. In both devices, the pH was regulated at 7.2 using feedback control by the CO_2_ gas concentration. After 48 h, the cell aggregates were collected to determine the aggregate diameter and cell count. The ratio of the final cell number to the initial cell number after 48 h of preparation was determined by dividing the cell number after 48 h of aggregate formation by the number of inoculated single cells into aggregate formation.

### 2.4 Medium exchange process using hollow fiber membrane separation

Medium exchange was performed using a Cell Washing Concentration System (Kaneka Corp., Japan) connected to the bioreactor via an aseptic connection (Figure 2Aa).According to the manufacturer’s instructions, hollow fibers with an inner diameter of 0.6 and 1.2 mm were primed with PBS before use. The circulation flow rate was set at 4.5 × 10^2^ mL/min, and the permeation flow rates were set at 0.5 × 10^2^ and 1.0 × 10^2^ mL/min. After the concentration operation, the fresh medium container was connected to the bioreactor system using the aseptic connector, and the working volume was set. The backwash was performed in a 10 L culture system, with a buffer container in the permeate line and a waste container in the circulation line. The timing of backwash was set when the permeate flow rate dropped below 85% of the set value. Cell aggregates were collected before and after medium exchange to determine aggregate diameter and cell count. The recovery rate (*R*) was calculated according to Equation 1 based on the number of input cells before the medium exchange process (*N*_input_) and the number of recovered cells after the medium exchange process (*N*_output_).

$$R=\frac{N_{\text{output}}}{N_{\text{input}}}$$ (1)

### 2.5 Sub process for medium preparation

The medium was prepared using a cell-processing isolator (CPi; Shibuya Corp., Japan). Cpi consists of a double-pass box and critical processing zone (Supplementary Figure S1A). Before Cpi was used, vaporized hydrogen peroxide decontamination (Shibuya Corp., Japan) was performed according to the manufacturer’s instructions. A material transfer procedure was established according to the contamination risk of the different packaging formats (Supplementary Figure S1B). Briefly, non-packaging materials (basal medium and supplements) were wiped with a disinfectant (Actril Surface Disinfectant; Cantel Medical Corp., USA) and brought into the first pass box. In this area, the surfaces of the materials were wiped with disinfectant, then sprayed with 70% ethanol (Ecolab Inc., USA), and then delivered to the second pass box. In the second pass box, the materials were wiped with 70% ethanol in one direction and then these were delivered to the cell processing area. Double-packed filling containers, along with double-packed cap assemblies consisting of tubing and aseptic connectors (AQG17004 AseptiQuik^®^ Sterile Connector; Colder Products Company, USA), were wiped with a disinfectant and delivered to the critical processing zone. In the case of primary or secondary packaging materials, after wiping with 70% ethanol in one direction in the first pass box, the outer packaging of materials was unpacked, and only the interior was delivered to the second pass box. In the second pass box, materials with only the content were wiped with 70% ethanol in one direction before being delivered to the critical processing zone. The primary packaging materials were unpacked in the second pass box, and only the interiors were delivered to the critical processing zone. In the critical processing zone, basal medium and supplements were added to the container. After being filled, the containers were capped with aseptic connectors.

### 2.6 Cell expansion process of hiPSC aggregates in 1 L bioreactor

Suspension culture was performed in a 1 L bioreactor (ZACROS Corporation Japan, working volume: 1.0 L) (Figure 2Ab) using a plastic fluid medium. Plastic fluid medium was prepared and supplied by the culture medium manufacturer, consisting of StemFit AK02N medium containing 0.02% polymer solution (FCeM-series FP001 Solution; Nissan Chemical Industries Ltd., Japan). Aggregates formed in a 100 mL delta paddle stirring device were resuspended in a plastic fluid medium and filled into a container with an aseptic connector. This container was aseptically connected to a 1 L bioreactor, and the entire volume was inoculated using a tubing pump at *t* = 0 h. The incubation was performed at 37 °C with 5% CO_2_; the cultures were incubated until *t* = 96 h under the condition of intermittent agitation for 5 min every 175 min at 100 rpm. As a control culture, aggregates were inoculated into a 30 mL delta paddle bioreactor (Able, Co., Japan), and incubated at 37 °C with 5% CO_2_ with continuous agitation at 55 rpm. Medium exchange was performed at *t* = 48 h using the method described above. Aggregates were collected to determine the aggregate diameter and cell number.

### 2.7 Cell expansion process of hiPSC aggregates in 10 L bioreactor

The suspension culture was performed in a 10 L bioreactor (ZACROS Corporation Japan, working volume: 10.0 L) (Figure 2Bb) using a plastic fluid medium. Aggregates formed in the large device were resuspended in a plastic fluid. Then, a 5 L delta paddle stirring device was aseptically connected to a 10 L bioreactor (Figure 2Bc), and the entire volume was inoculated using a tubing pump at *t* = 0 h. The container of fresh medium was aseptically connected to a 10 L bioreactor, and the fresh medium was pumped to a total volume of 10 L. The incubation was performed under 37 °C with 5% CO_2_; the cultures were incubated until *t* = 120 h under the condition of intermittent agitation for 20 s every 240 min at 65 rpm. The dissolved oxygen concentration was monitored throughout the culture using a polarographic oxygen sensor (Mettler Toledo, Switzerland), with measurements taken immediately following each agitation event. Medium exchange was performed at *t* = 48 h and 96 h using the method described above. Aggregates were collected to determine the cell counts. The aggregates were collected at *t* = 120 h and washed with PBS. The dissociation reagent (Accumax: Innovative Cell Technologies Inc., USA) was applied for 10 min at 37 °C, and hiPSC aggregates are dissociated into single cells. The collected cells were used for flow cytometric analysis and a trilineage differentiation potential assay.

### 2.8 Determination of cell number and aggregate diameter

To determine cell number, collected cell aggregates were washed with PBS and dissociated into single cells using Accumax with 10 µM ROCK inhibitor. Cells were counted with an automated cell counter (TC20; Bio-Rad Laboratories Inc., Hercules, CA, USA) using the trypan blue exclusion method. Hereafter, the term ’cell number’ refers to the number of viable cells. The apparent specific growth rate (*μ* ^app^) was calculated according to Equation 2, where *N*_t_ represents the cell number at time t h, and Δt is the time interval between measurements.

$$\mu^{\text{app}}=\frac{\ln\left(N_{t+\Delta t}/N_{t}\right)}{\Delta t}$$ (2)

To determine the aggregate diameter, images were captured using a phase-contrast microscope (CKX53; Olympus Co., Japan). The projected area *S*, µm^2^, was measured by ImageJ, and the equivalent surface diameter (*D*) was calculated according to Equation 3.

$$D=\sqrt{\frac{S}{\pi }}$$ (3)

The coefficient of variation (CV) of the aggregate diameters was calculated for all samples.

### 2.9 Quantification of pluripotency

To elucidate the undifferentiated state of hiPSCs, the expression of pluripotency markers Oct3/4, SSEA-4, and TRA1-60 was assessed by flow cytometry. Dissociated cells were fixed and permeabilized using a Cytofix/Cytoperm Permeabilization Kit (BD Biosciences, USA) and stained with the following markers: Oct3/4 (PE-conjugated human/mouse Oct3/4 Fluorescein; BD Biosciences, USA), SSEA-4 (FITC-conjugated human/mouse SSEA-4 fluorescein; R&D Systems Inc., USA), and TRA-1-60 (FITC-conjugated human/mouse TRA-1-60 fluorescein; Merck, Germany). Stained cells were measured using a flow cytometer (CyFlow Cube 6; Sysmex Co., Japan), and the obtained data were analyzed using an analysis software (FlowLogic; Inivai Technologies, Australia).

### 2.10 Assay for trilineage differentiation potential

At *t* = 120 h, the dissociated cells were re-seeded onto an iMatix-511-coated polystyrene culture surface and cultured according to the manufacturer’s instructions using a commercially available trilineage differentiation kit (Stemdiff™ trilineage differentiation kit; Stem Cell Technologies, Canada) and an undifferentiated medium: StemFit AK02N. The cultured cells were washed with PBS and fixed with 4% paraformaldehyde (Fujifilm Wako Pure Chemical Industries, Japan) for 10 min at room temperature. After washing with PBS, the cells were permeabilized with PBS containing 0.5% Triton X-100 (Fujifilm Wako Pure Chemical Industries, Japan) for 5 min. After another wash, non-specific proteins were blocked with Block Ace (Dainippon Sumitomo Pharma Co., Ltd., Japan) for 90 min at room temperature. The cells were then incubated overnight at 4 °C with following primary antibodies in PBS containing 10% Block Ace: anti-OCT3/4 (Santa Cruz Biotechnology), Anti-PAX6 (Sigma-Aldrich, USA), Anti-Brachyury (Santa Cruz Biotechnology, USA), and Anti-SOX17 (Cell Signaling Technology Inc., USA). After washing with Tris-buffered saline, the cells were incubated with an Alexa Fluor 488-conjugated secondary antibody (AF488 goat anti-rabbit; Thermo Fisher Scientific) for 60 min at room temperature. Nuclei were stained with 4′,6-diamidino-2-phenylindole (DAPI; Thermo Fisher Scientific). Finally, the immunofluorescence images were captured using an imaging system (IN Cell Analyzer 2000; Cytiva, USA) with a ×10 objective lens.

### 2.11 Statistical analysis

Quantitative data are expressed as mean ± SD obtained from three independent experiments, including 1 L and 10 L bioreactor performances. Statistical tests were performed to analyze the differences between the two experimental groups using the unpaired Student’s t-test. Differences were considered statistically significant at *p* value of less than 0.05.

## 3 Results

### 3.1 Process parameter evaluation at small scale

**FIGURE 3:**
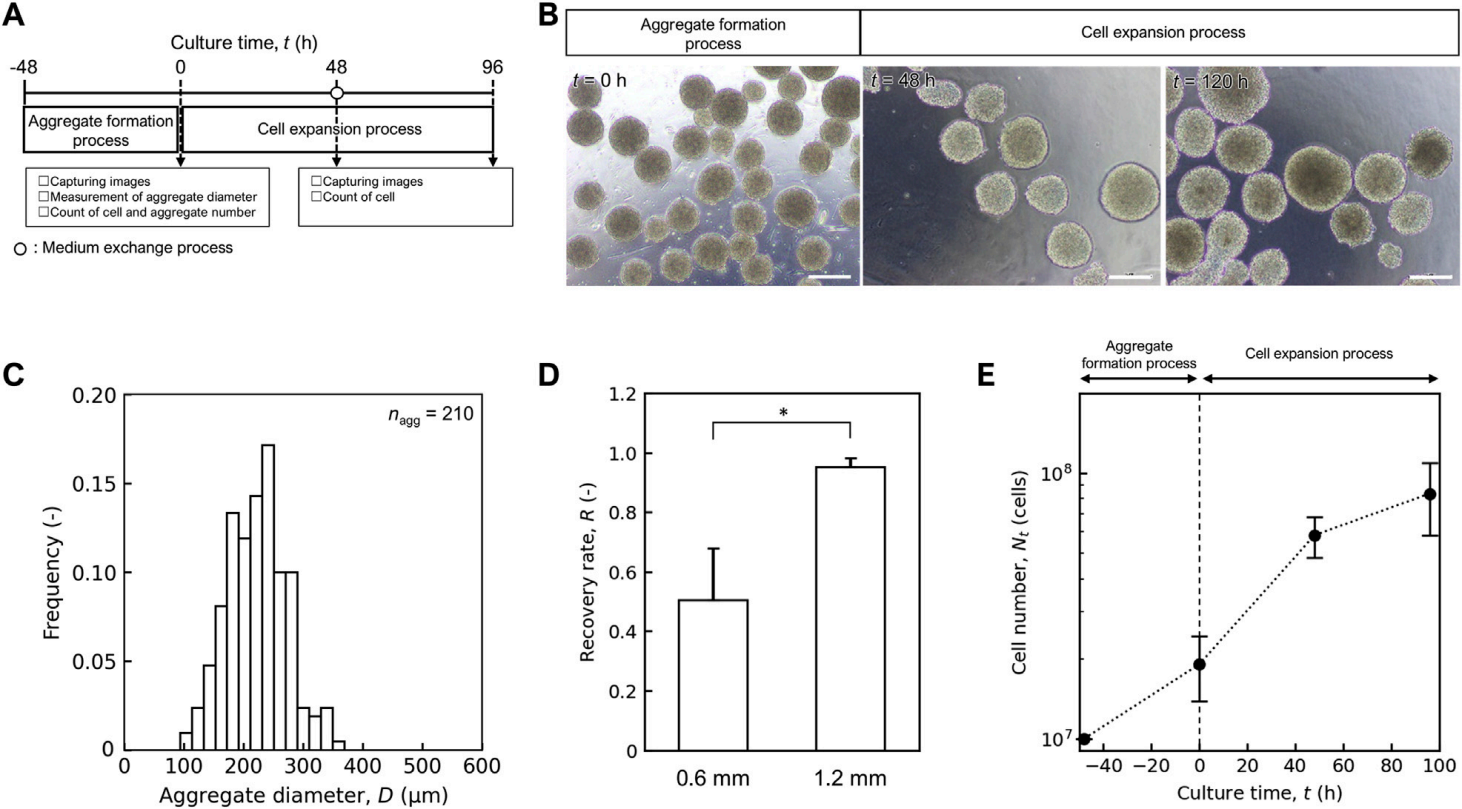
Performance of mass culture system for small scale. **(A)** Schematic diagram of hiPSC mass culture system for small scale. **(B)** Representative images of aggregate in aggregate formation and cell expansion process. Scale bars: 200 µm. **(C)** Distribution of aggregate diameter after aggregate formation process at *t* = 0 h. Sample size are shown as *n*_agg_. **(D)** Comparison of recovery rate (*R*) of medium exchange process with inner diameter of 0.6 and 1.2 mm. Error bars represent the standard deviation (*n* = 3). Statistical significance was determined using Student’s t-test (*p* < 0.05). **(E)** Time profile of cell number *N*_t_ (cells). Error bars represent the standard deviation (*n* = 3).

As a foundation for the mass culture system design, we established a hiPSC culture system for small-scale evaluation of its capability. The culture system was tentatively designed as shown in Figure 3A, and the parameters were evaluated for each unit process. First, two types of agitation systems were compared during the aggregate formation process: orbital shaking and stirring using a delta paddle. Dissociated single hiPSCs were inoculated into aggregate formation devices. In Erlenmeyer flasks and the 1 L bioreactor with orbital shaking, many single cells and large aggregates were observed (Supplementary Figure S2). In the case of the stirring method, spherical aggregates were observed at *t* = 0 h (Figure 3B), and the average aggregate diameter and the coefficient of variation were (2.22 ± 0.49) × 10^2^ μm and 0.22 respectively (Figure 3C). The ratio of final (*t* = 0 h) to initial (*t* = −48 h) cell number was 1.97 ± 0.15, and cell loss was prevented during aggregate formation.

In the medium exchange process, a TFF system with hollow fiber membranes was selected to concentrate the aggregates. The aggregate-suspended culture medium was circulated through the lumen of the hollow fibers, and only the medium permeated through the membrane wall to the outer side. The balance between the circulation and permeation flow rates and the inner diameter of the lumen was considered to achieve a high recovery rate (*R*). The ratio decreased with increasing permeate volume at a flow rate of 1.0 × 10^2^ mL/min (Supplementary Figure S3A) and clumps of cell aggregates were observed inside the lumen of the hollow fiber membrane (Supplementary Figure S3B). In contrast, at a flow rate of 0.5 × 10^2^ mL/min, *R* was maintained even when the permeate volume was increased. We also compared *R* using hollow fibers with different inner lumen diameters (0.6 and 1.2 mm) for a 1 L medium exchange. The *R* value for 1.2 mm inner diameters (0.95 ± 0.03) was significantly higher than that for 0.6 mm diameter (0.50 ± 0.17) (Figure 3D, *p* < 0.05). Based on these results, we selected a 1.2 mm inner diameter of hollow fiber with a permeate flow rate of 0.5 × 10^2^ mL/min for subsequent culture experiments.

During the cell expansion process, we evaluated the capacity of an intermittent agitation culture system using plastic fluid in a 1 L bioreactor designed to maintain the aggregate structure and proliferation potential. Aggregates formed in a 100 mL delta paddle stirring device were suspended in plastic fluid medium and inoculated into a 1 L bioreactor at *t* = 0 h with a cell number of (1.91 ± 0.53) × 10^7^ cells. Medium exchange was performed at *t* = 48 h using a hollow-fiber membrane with an inner diameter of 1.2 mm. By *t* = 96 h, the cell number was increased to (8.86 ± 0.32) × 10^7^ cells (*n* = 3) (Figure 3E), and the apparent specific growth rate, *μ* ^app^ in the cell expansion process was (1.53 ± 0.07) × 10^–2^ h^-1^, which was comparable to that observed in conventional stirred bioreactor used for hiPSC culture ((1.98 ± 0.20) × 10^–2^ h^-1^). The designed culture system and process parameters were scaled up to a 10 L system.

### 3.2 Performance of 10 L mass culture system

**FIGURE 4:**
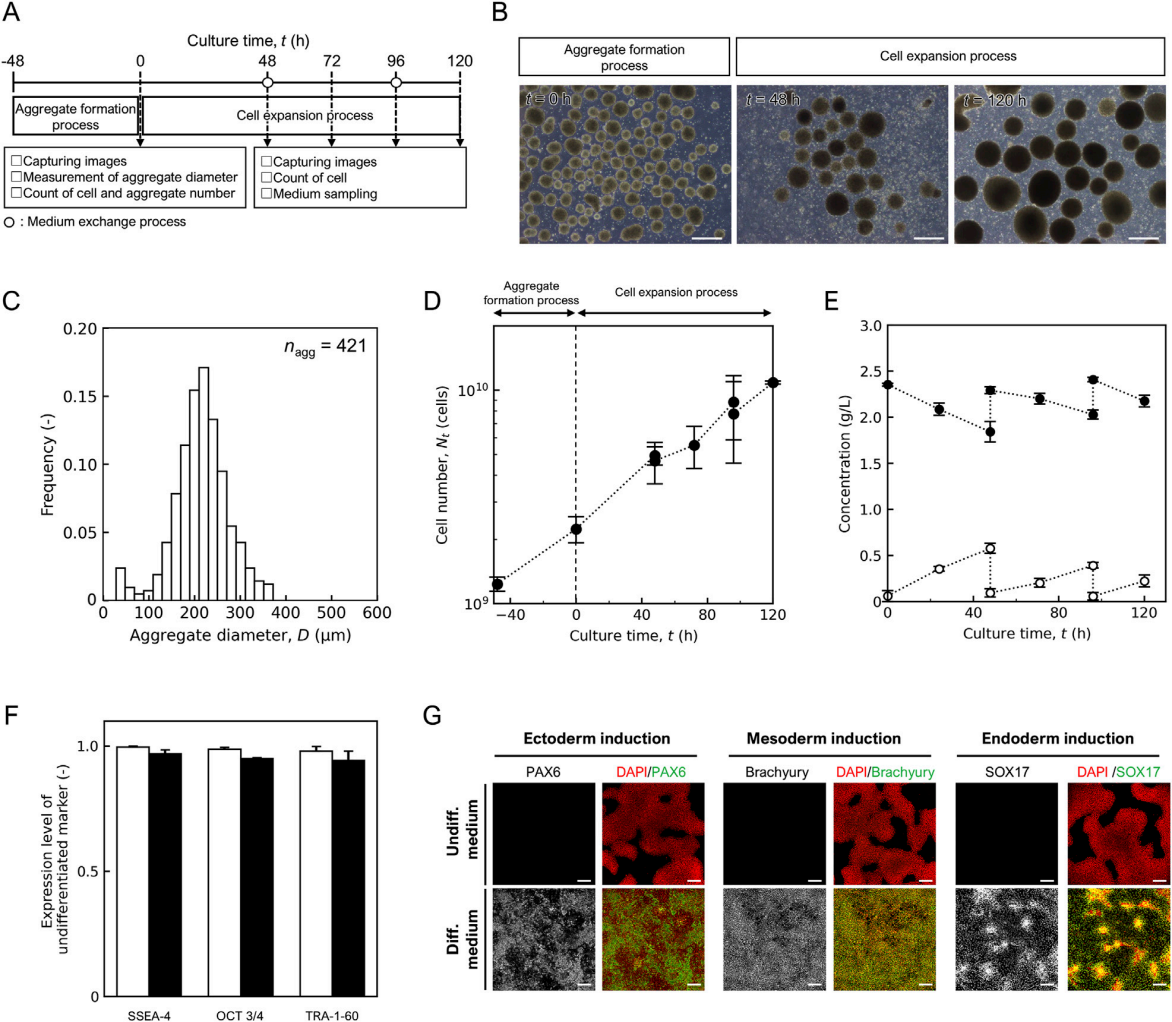
Performance of 10 L mass culture system. **(A)** Schematic diagram of hiPSC mass culture system. **(B)** Representative images of aggregate in aggregate formation and cell expansion process. Scale bars: 500 µm. **(C)** Distribution of aggregate diameter after aggregate formation process at *t* = 0 h. Sample size are shown as *n*_agg_. Time profile of **(D)** cell number *N*_t_ (cells) and **(E)** concentration of lactic acid (open) and glucose (closed). **(F)** Flow cytometry analysis to quantify pluripotency marker expression, open, *t* = −48 h; closed, *t* = 120 h. **(G)** Fluorescence staining images of differentiation markers (PAX6, brachyury, and SOX17) expression in cells maintained in undifferentiated medium and differentiation medium. Scale bars, 200 µm. Error bars represent the standard deviation (*n* = 3).

By combining the individual unit processes and scaling up, we performed independent 10 L mass cultures three times, as shown in Figure 4A. During the aggregate formation process, cells were cultured in a large 5 L device. Spherical aggregates were observed at *t* = 0 h (Figure 4B), and the average diameter and the coefficient of variation were (2.14 ± 0.60) × 10^2^ μm and 0.28, respectively (Figure 4C). The ratio of final (*t* = 0 h) to initial (*t* = −48 h) cell number was 1.99 ± 0.28. There were no significant differences in the average aggregate diameter and ratio of cell numbers between the different scales, and the aggregate formation capacity was comparable for scaling up.

In the cell expansion process, the aggregates were suspended in a fresh plastic fluid medium and inoculated at *t* = 0 h at a concentration of (2.24 ± 0.31) × 10^9^ cells/L into a 10 L bioreactor in a closed system. The shaking rate was set at 65 rpm by Froude number-based scaling-up for orbital shaking bioreactor (Tissot et al., 2010), and intermittent agitation time was set for 20 s every 240 min for maintenance of oxygen concentration above 0.68 mg/L which was critical for hiPSC growth (Supplementary Figure S4). In this intermittent culture system without the addition of the ROCK inhibitor, cell density decreased over time (Supplementary Figure S5A), and single cells were observed more frequently at *t* = 48–120 h (Supplementary Figure S5B). Conversely, when cultured in a plastic fluid medium containing 20 µM ROCK inhibitor, which promotes ECM secretion and structural changes that suppresses apoptosis caused by aggregate collapse, the number of observed single cells tended to decrease (Figure 4B), and the cell number increased over time (Figure 4D). In three independent trials, all showed similar growth trends, and the cell number reached (1.09 ± 0.02) × 10^10^ cells at *t* = 120 h (*n* = 3). The apparent specific growth rate, *μ* ^app^ was (1.33 ± 0.12) × 10^–2^ h^-1^. The lowest dissolved oxygen concentration observed during the culture period was 1.23 mg/L. During the long-term concentration of the medium exchange process, the permeate flow rate decreases as the volume of the extracted medium increases. A backwash was performed when the permeation rate dropped below 85% and the permeate rate recovered to 97% ± 2% of the set value (Supplementary Figure S6A). Numerous pieces of debris were observed in the backwash waste solution (Supplementary Figure S6B). Lactate and glucose concentrations were refreshed after medium exchange (Figure 4E). The lactate concentration was below 1.0 g/L throughout the culture period and the exchange efficiency was 0.84 ± 0.10.

The expression of the undifferentiated markers SSEA4, Oct3/4, and TRA-1-60 was maintained before and after culture at *t* = 120 h (Figure 4F). To confirm the trilineage differentiation potential, harvested hiPSCs were cultured in a differentiation medium. After differentiation, the cells expressed PAX6, Brachyury, SOX17, indicating trilineage differentiation (Figure 4G).

## 4 Discussion

### 4.1 Aggregate formation process and cell expansion process for hiPSCs

In this study, we developed a mass-culture system for hiPSCs based on cell manufacturability. Because of the anchorage dependency, the first step of this system focused on the formation of homogeneous aggregates. While orbital shaking resulted in the formation of coalescent aggregates (Supplementary Figure S2) due to the “Einstein’s tea leaf paradox” flow pattern (Yamamoto et al., 2018), our delta paddle stirring method demonstrated the medium flowed laterally at a constant rate (Matsuura et al., 2012). This method can be used to easily prepare homogeneously sized aggregates with a scale-up. Prepared mean aggregate diameter are approximately 220 μm, with a coefficient of variation that is less than 0.3. Abbasalizadeh et al. proposed that aggregates with a diameter of about 190–215 µm are suitable for cell proliferation for hiPSC expansion culture (Abbasalizadeh et al., 2012). The aggregate diameters prepared in this study were suitable for subsequent cell expansion.

The successful formation of homogeneous aggregates with an appropriate size distribution provided an optimal starting point for the subsequent cell expansion process. In the next step, the maintenance of aggregate structures, which directly affects both aggregate integrity and growth potential, becomes a critical challenge for scalable cell expansion. In this study, a plastic fluid was used as a culture system to supply a suitable environment. Oxygen supply is critical for scale-up. Under static conditions, the diffusion capacity at the gas-liquid interface was quite low compared to that under dynamic conditions. In a previous study, hiPSC aggregates could not grow when the specific gas-liquid surface area was 8.1 × 10^−2^ m^2^/m^3^ without sparging (Yamamoto and Kino-oka, 2021). Because this 10 L scale culture system has a similar specific surface area (9.6 × 10^−2^ m^2^/m^3^), an oxygen supply method is needed for culture system design. However, agitation induces hydrodynamic stress, which is the primary cause of cellular damage (Papoutsakis, 1991; Elias et al., 1995; Mollet et al., 2007; Hu et al., 2011). As this stress easily increases with scale-up, management of flow characteristics is important. In this study, we employed an intermittent agitation culture system to supply oxygen and suppress hydrodynamic stress using the thixotropy of this fluid. The agitation period was narrowed to maintain a sufficient oxygen concentration for cell growth. The relationship between dissolved oxygen concentration and specific growth rate followed Michaelis-Menten kinetics, and hiPSC growth was not inhibited at oxygen concentration above 0.68 mg/L (Supplementary Figure S4). Intermittent agitation maintained the dissolved oxygen concentration above 1.23 mg/L during culture, suggesting that oxygen was also efficiently supplied to this system. Intermittent agitation has previously reported in hiPSC culture, primarily to provide mechanical cues to enhance differentiation to cardiomyocytes (Correia et al., 2014; Ting et al., 2014). However, applying such a strategy for expansion in conventional Newtonian fluids has a significant challenge, as the long static periods would lead to aggregate sedimentation and coalescence. Our approach lies on the combination of this agitation strategy with a plastic fluid. The fluid’s yield stress prevents sedimentation, thereby enabling a low-shear environment for large-scale expansion that is not achievable with intermittent agitation alone. To design the agitation rate, dimensionless quantities that predict flow patterns were used as scale-up parameters. The Reynolds number, which represents the ratio of inertial forces to viscous forces, indicates the flow patterns at the tip of the impeller and has been used as a standard for the scale-up of stirred tank reactors (Imamoglu and Sukan, 2013). For scale-up, we initially used the Froude number as an scale-up indicator (Tissot et al., 2010), but hiPSC aggregates collapsed despite this approach (Supplementary Figure S5B). This suggests that the conventional scale-up criteria may be difficult to apply to the aggregate culture of hiPSCs. In previous animal cell culture systems, shear stress was reported to be the main cause of cell damage, compared to normal stress. In the case of aggregate culture, normal stress caused by the flow or their collisions applies pressure towards the center of the aggregates and may cause aggregate collapse, leading to apoptosis. The influence of normal stress on the total stress increases with increasing rotation speed in the reactors (Eslahpazir Esfandabadi et al., 2012), and is assumed to have a similar effect on scale-up. This suggests that further studies on the hydrodynamics of aggregate culture systems are required. Additionally, the robustness of hiPSC aggregate structures should be improved as an alternative approach to culture system design. In this study, a ROCK inhibitor was used to improve the robustness of the culture environment. Exposure to ROCK inhibitors increases ECM secretion to maintain the aggregate structure (Kim et al., 2018) and alters the fibril structure of collagen type I at the periphery of the aggregates (Matsumoto et al., 2022). In our study, exposure to the ROCK inhibitor prevented the disruption of aggregates and maintained a specific growth rate (Figures 4B,D). This suggests that the change in ECM structure improves tolerance to hydrodynamic stress and inhibits the disruption of aggregates. The mechanical forces experienced in different culture conditions can influence the epigenetic memory and pluripotent state maintenance of hiPSCs (Thanuthanakhun et al., 2021), highlighting the importance of controlling mechanical environments in aggregate culture. These biological and engineering approaches using plastic fluids can achieve 10^10^ hiPSC culture on a 10 L scale. To scale up to a larger scale, an additional approach for understanding the hydrodynamics using techniques such as computational fluid dynamics (CFD) should be considered (Borys et al., 2019).

### 4.2 Medium exchange process for hiPSC aggregates using hollow fiber membrane

During the medium exchange process using the hollow fiber membranes, two types of blockages were observed. The first is the membrane surface blockage, which is a typical problem in membrane separation (Clincke et al., 2013; Walther et al., 2019). The backwash removed the cake composed of smaller particles (Supplementary Figure S6B). They resembled the cell debris generated during the culture period. Unlike dead cells, these disrupted cells cannot be identified by the trypan blue exclusion method; however, hydrodynamic stress has the potential to break up cell membranes and induce necrosis (Regmi et al., 2017). Separation by gravity is primarily used in small-scale cultures. Using this method, most of the smaller particles remained in the supernatant and were removed without consciousness. Their contamination may cause safety issues in clinical treatment, and consideration of debris management is necessary when scaling up manufacturing. Another blockage was observed when the cell aggregates formed clumps in the hollow fibers and blocked the flow paths (Supplementary Figure S3B). The spaces inside conventional hollow fibers designed for single cells are too narrow for cell aggregates. The recovery rate increased with the inner diameter of the hollow fibers. The recovery rate was also improved by decreasing the permeate flow rate at a constant circulation flow rate. The average linear velocities of tangential flow are 5.9 and 6.3 cm/s when the permeate flow rates are 1.0 × 10^2^ and 0.5 × 10^2^ mL/min, respectively. In this case, the average linear velocities in the vertical direction are 2.2 × 10^−3^ and 1.1 × 10^−3^ cm/s, suggesting that lower velocities reduce the probability that cell aggregates will contact and trap the membrane and decrease a risk of clumps formation. In contrast, the permeation flow rate directly affects the operating time of the medium exchange process. If we employ a hollow fiber system with a 0.5 × 10^2^ mL/min permeation rate, it takes at least 160 min to concentrate cell suspension from 10 L to 2 L for 80% exchange of medium. Long-term deviation from a stable culture environment may have a negative effect on cells. In this study, we employed two of these systems in parallel and the deviation time was reduced to 100 min. When scaling up to a larger scale, increasing the membrane surface area and designing hollow fibers with larger inner diameters are required to reduce clump formation and increase processing volume.

### 4.3 Medium preparation process for 10 L scale culture

Vapor hydroperoxide decontamination is a standard method used to ingress materials into isolator systems (Hubbard et al., 2018). The maximum load for a single decontamination cycle must be established through validated processes under the worst-case conditions. Generally, an excessive material load can obstruct vapor diffusion, resulting in shadowed areas where the decontaminant fails. This means that there is a risk of preparing large amounts of culture medium at once. As this method requires 6–8 h (Hubbard et al., 2018), several separate loads are not suitable for preparation at the time of use. In this culture system, we used a flexible transfer method with a double-pass box isolator system and risk-based installation according to the contamination risk with different packaging formats. This method takes 7.3 h for material transfer and 0.9 h for the filling operation and reduces the time required for the medium preparation processes. To further decrease the time required for material transfer, the packaging format should be standardized and fitted to simplify the transfer operation, which is essential for supplier cooperation. This suggests the need for an alternative preparation process that can be scaled up to larger volumes. Medium preparation for a 1000 L-scale culture of animal cells is commonly performed using soluble powder medium and filtration for sterilization. Because this simple preparation is scalable, the development of powder technologies for the solidification of media containing various high-molecular-weight proteins is required.

### 4.4 Scaling-up design for hiPSC aggregate culture system

Based on the concept of cell manufacturability, the considerations of mass culture systems for hiPSC aggregates differ from those of conventional anchorage-independent cells. Recent studies require highlight complementary principles for resolving this challenge. For instance, Borys et al. (2024) have emphasized the importance of designing for process robustness, using a framework of process input and output variables to evaluate how a culture system performs over long-term serial passaging. Our study addresses the design and integration of the culture system to enable spatial scale-up to the 10 L level. Our approach guided deconstruction of overall process into distinct unit process and the specific engineering solutions for each unit processes.

Unlike traditional approaches where cells are directly inoculated into small to bioreactors, hiPSCs require specific operations because dissociated cells easily undergo apoptosis in suspension. Most conventional studies have directly inoculated dissociated hiPSCs into bioreactors, and the size and efficiency of cell aggregates have been controlled by examining agitation conditions in various bioreactors (Meng et al., 2017; Dang et al., 2021; Torizal et al., 2022; Ueki et al., 2024). This suggests that the scalability of seed trains in various bioreactors is influenced by their agitation systems. While achieving the 10 L scale has been a significant challenge, several studies have recently reported success using different culture systems. The Vertical-Wheel^®^ bioreactor, for example, is designed to create a more homogeneous and low-shear hydrodynamic environment. While its performance has been experimentally demonstrated up to the 0.5 L scale (Cuesta-Gomez et al., 2023), CFD analysis has been used to predict its scalability up to 15 L (Dang et al., 2021). Meanwhile, microencapsulation technology physically protects cells from hydrodynamic stress (Fattahi et al., 2021), a strategy has enabled remarkable expansion rates of over 275-fold in a 10 L bioreactor (Cohen et al., 2023). However, while highly effectiveness, the manufacturing workflow must incorporate an additional encapsulation step, and depending on the final application, a subsequent decapsulation stem may be required to harvest cells, adding complexity to the overall process.

These systems represent significant advances in the field. The intermittent agitation culture system using a plastic fluid presented in this study offers another original approach to overcoming these same challenges. Our results, which demonstrate the production of over 10^10^ cells, indicate that this novel system has a mass culture potential comparable to these leading alternative systems, thereby providing a valuable new platform for industrial scale hiPSC manufacturing. However, looking forward toward future scalability beyond the 10 L scale presents additional challenges that require a deeper understanding of the process. As our findings suggest, conventional flow analysis targeting shear stress is not sufficient, and that it is necessary to consider multiple factors, including normal stress, for the maintenance of the aggregate structure. While oxygen is commonly supplied from the upper surface, it limits the scale-up. In this study, upper surface aeration was employed, which was sufficient for a 10 L scale, and a plastic fluid culture system had the potential to supply oxygen by bubble sparging (Yamamoto and Kino-oka, 2021). Therefore, a combination of these culture systems may be applicable for scaling up to 10^2^ L.

In conventional systems, medium exchange is often performed via gravity separation to remove the supernatant. The requirement of aggregate culture is to maintain a small size because of contact inhibition and diffusion limitations; however, they take a longer time for sedimentation. Coalescence between hiPSC aggregates was induced within 40 min (Matsumoto et al., 2022), which suggests that the employed sedimentation-based concentration systems for medium exchange are related to their scalability. When considering the scale-up of the medium exchange process, continuous centrifugation or membrane separation with a tangential flow may be suitable for preventing coalescence with liquid flow. However, because coalescence easily occurs even under flow conditions on the 0.6 mm inner diameter of the hollow fiber (Supplementary Figure S3), the consideration point of aggregate filtration is different from that of conventional single-cell culture. Therefore, additional systems are required for aggregate culture. This study designed a specific mass-culture system with a combination of several processes.

At the system level, the integration of these processes into a reproducible manufacturing system is a significant aim of cell manufacturability. While reproducibility is an important factor in manufacturing, the establishment of reproducible manufacturing processes for cell products is challenging because of the fluctuations of the cells themselves (Kino-oka et al., 2019). In this study, a scaling-up design for a hiPSC aggregate culture system was proposed, which contributes to the development of highly reproducible culture systems. The obtained hiPSC aggregates underwent enzymatic treatment, followed by a filling-freezing process. Assuming that all the cells are aliquoted and frozen at 10^6^ cells/vial, 10^4^ vials will be stored. As the suspension time in the cryopreservation solution affects the cell potential, Kagihiro et al. calculated the operation time in aliquots (Kagihiro et al., 2018). Based on this study, an operation time for aliquots is required to keep 1 × 10^4^ vials/h for maintaining cell potentials, which suggests the requirement for equipment design based on cell manufacturability in subsequent filling and freezing processes. These considerations for scaling up hiPSC culture systems highlight both the progress made in this study and the remaining challenges for further scale expansion.

## 5 Conclusion

In this study, we designed a 10 L mass culture system for hiPSC aggregates based on cell manufacturability. First, we proposed a design procedure for hiPSC aggregates. We designed four-unit processes on a small scale and developed a 10 L scale culture system. In this system, hiPSC aggregates were cultured under intermittent agitation by fluid plasticity, with medium exchange by hollow-fiber membrane filtration. By inhibiting the ROCK pathway, we formed cell aggregates that were more resistant to hydrodynamic stress and obtained 10^10^ cells in one batch. This culture system was designed as a closed system and an aseptic medium preparation process was established. This design was based on the principles of cell manufacturability, indicating the requirements for scaling up, and is expected to bridge the gap between research and clinical applications by providing a basis for the design of mass culture systems closer to a manufacturing scale.

## Data Availability

The original contributions presented in the study are included in the article/Supplementary Material, further inquiries can be directed to the corresponding author.
