# Supplementary material for: Development of 10 L mass culture system of human induced pluripotent stem cells with intermittent agitation using plastic fluid
Source: Front Bioeng Biotechnol. 2025 Nov 18;13:1664723. doi: 10.3389/fbioe.2025.1664723 (PMC12669166; doi:10.3389/fbioe.2025.1664723)
Supplement: Supplementary file 1 [file Supplementaryfile1.docx]

Supplementary Material


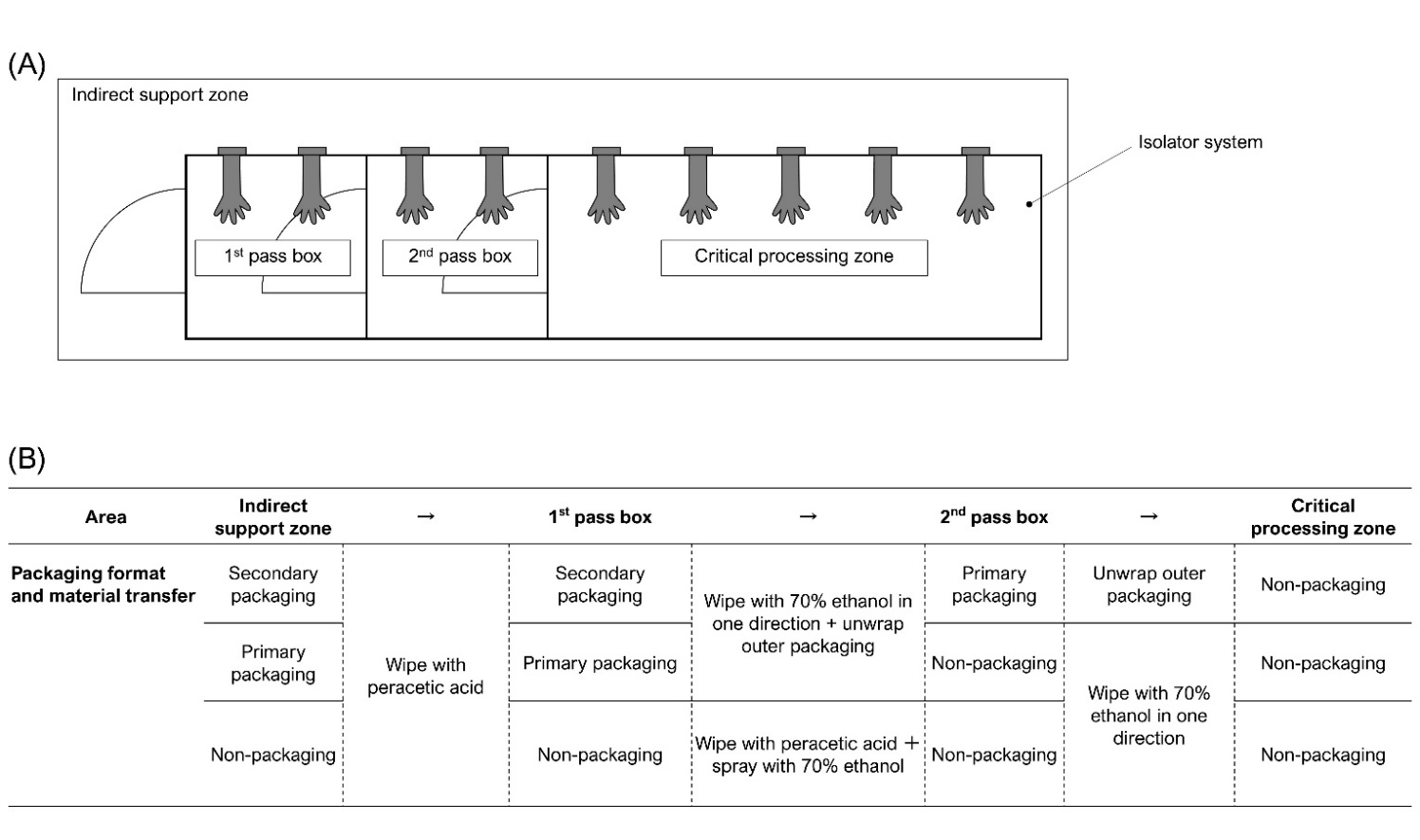


**Supplementary Figure 1.** Schematic images of isolator system and material transfer procedures. (A) Double-pass box isolator system was used for disinfection and aeration of materials to be transferred. (B) The material transfer was identified depending on the risk of contamination due to differences in the packaging format.

**
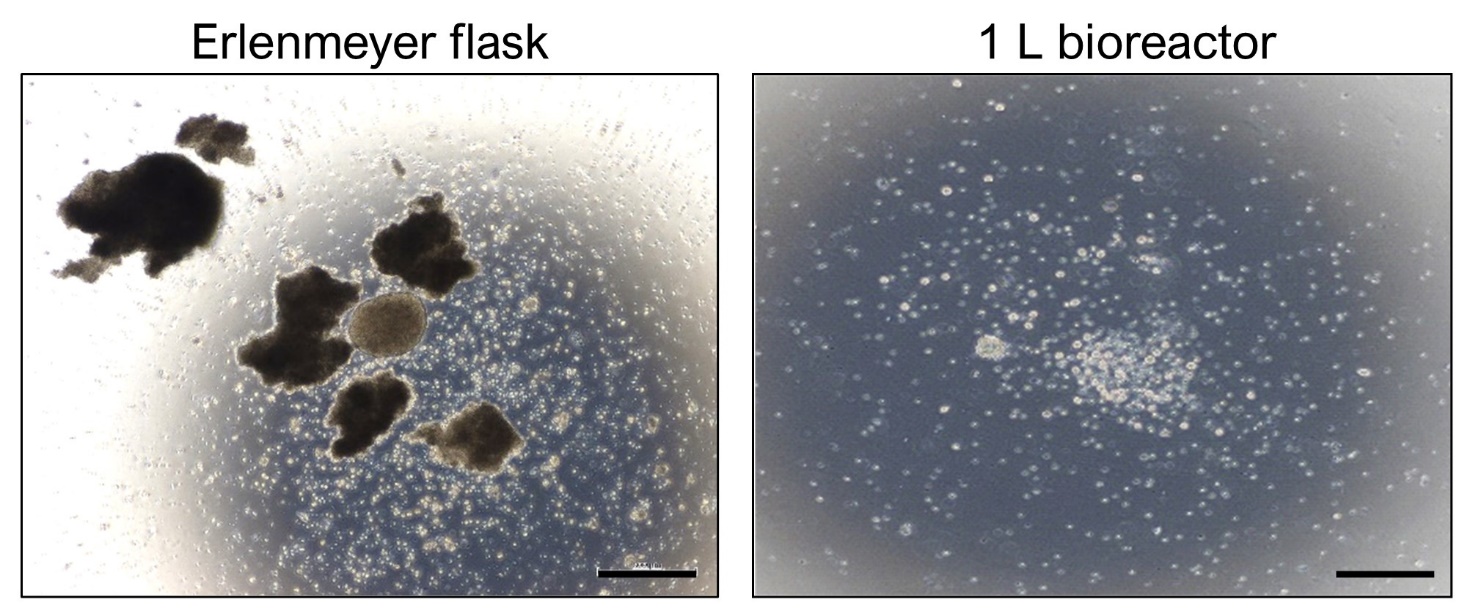
**

**Supplementary Figure 2.** Representative images of aggregates formed in the aggregate formation process using an orbital-shaker in 100 mL Erlenmeyer flask and 1 L bioreactor after 48 h of preparation. Scale bars, 200 µm.


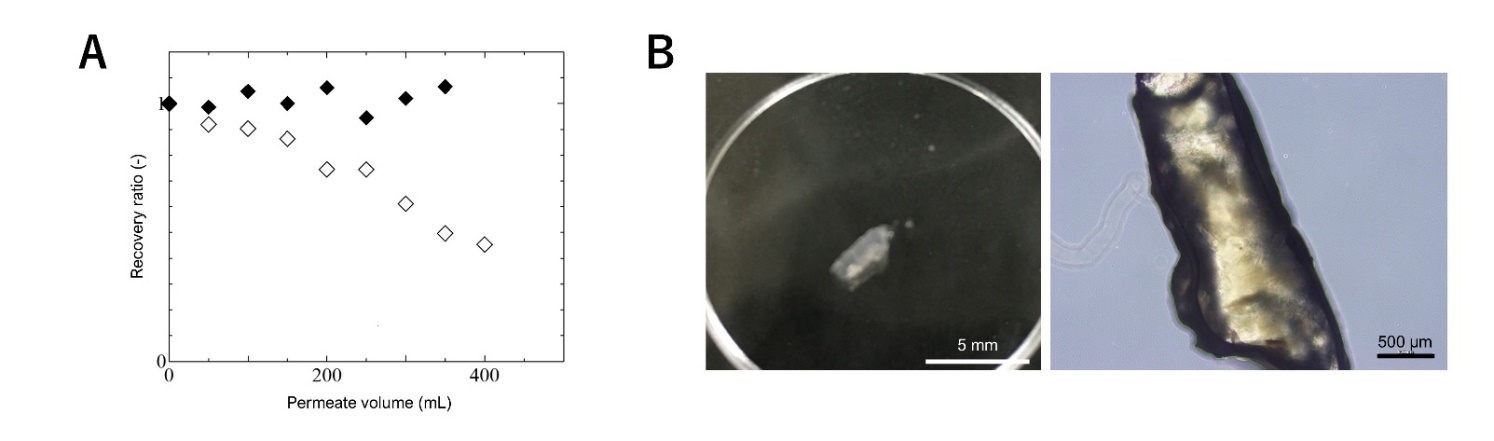


**Supplementary Figure 3.** Medium exchange process with TFF system using hollow fiber membranes. (A) Relationship between permeate volume and recovery ratio of aggregates concentration. Closed diamond: permeate flow rate was 0.5 × 10^2^ mL/min, Open diamond: permeate flow rate was 1.0 × 10^2^ mL/min (B) Representative images of clumps of aggregate clogged in the hollow fiber.


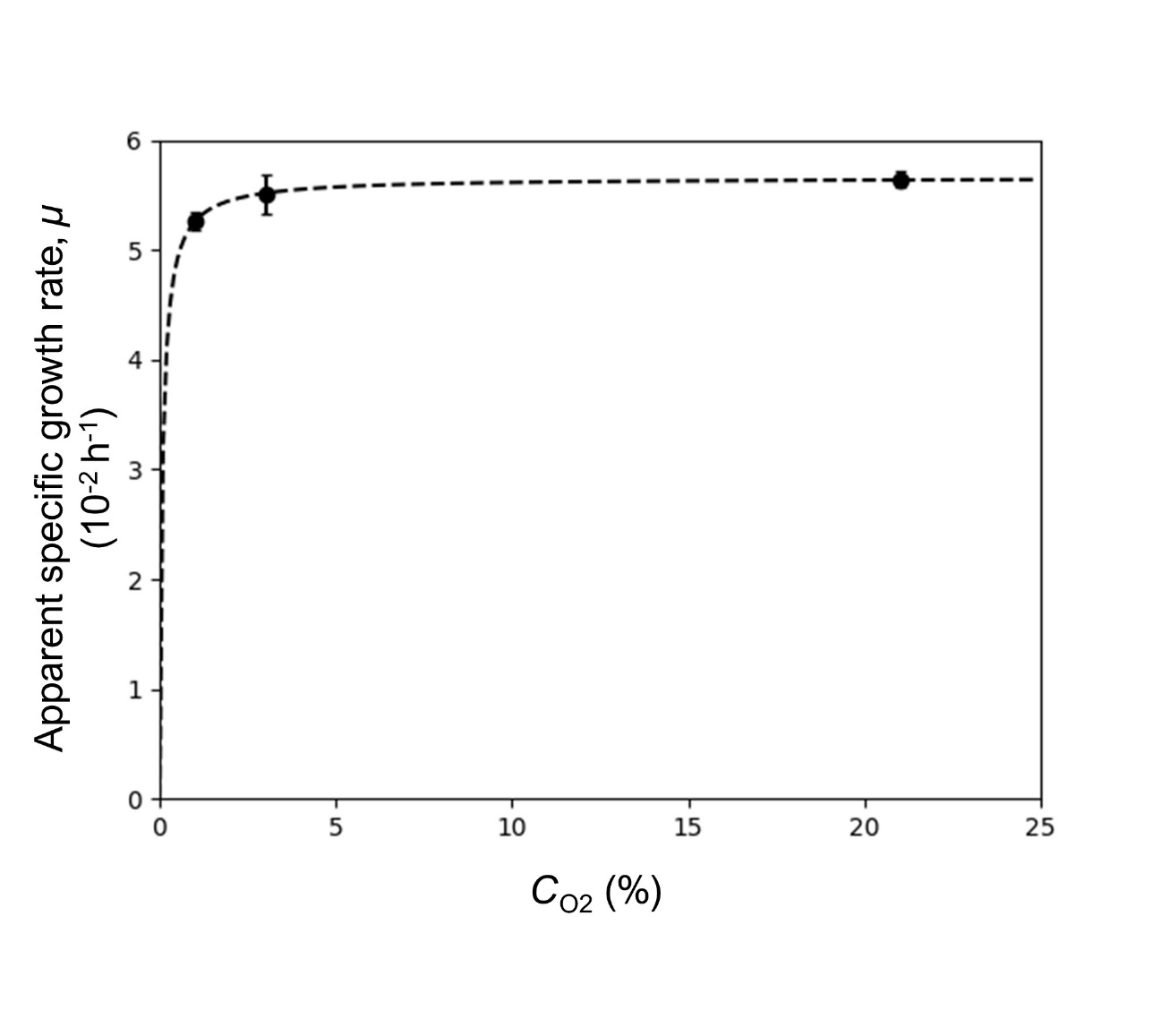


**Supplementary Figure 4.** Relationship between oxygen concentration and apparent specific growth rate in 2D static culture of hiPSCs. Oxygen concentrations, *C*_O2_ were set at 1, 3, 21 % in a hypoxic incubator. Apparent specific rates were determined for the culture period of 24-72 h (*n* = 3). The dashed line indicates the regression curve fitted to Michaelis-Menten equation; *µ* = 5.7 × 10^-2^ *C*_O2_ / (7.6 × 10^-2^ + *C*_O2_).


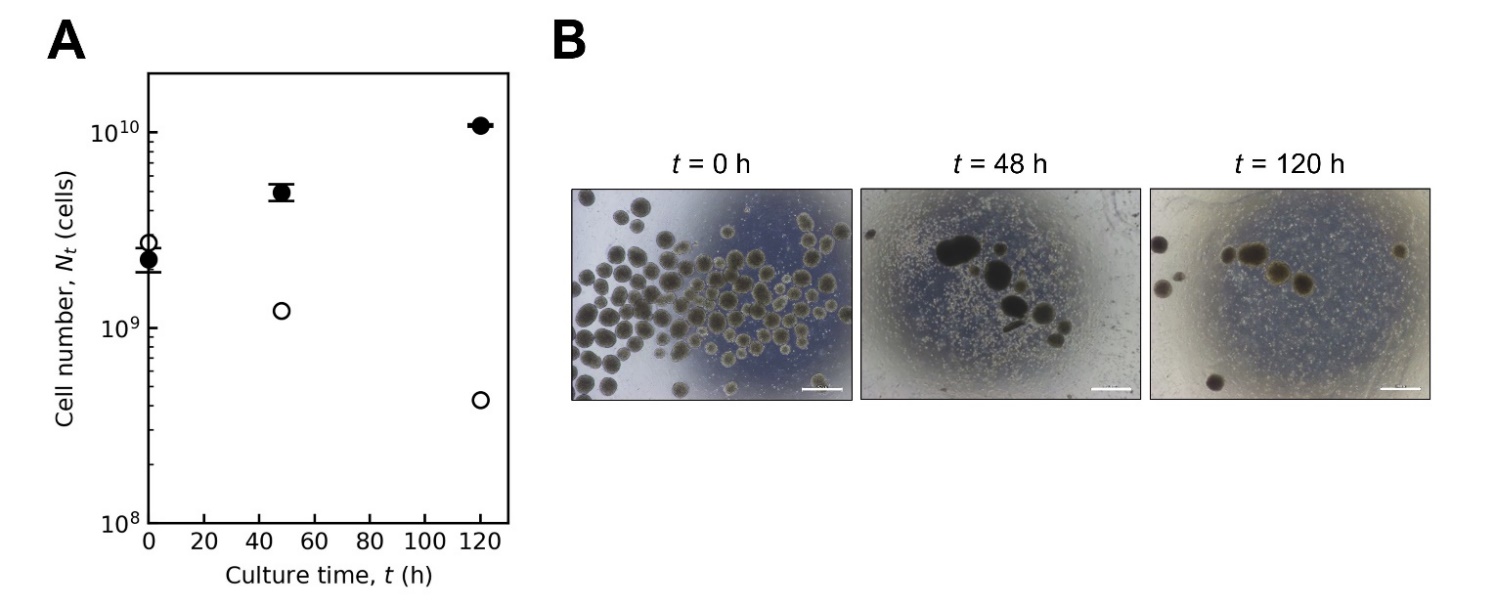


**Supplementary Figure 5.** Performance of 10 L mass culture for hiPSCs without ROCK inhibitor. (A) Growth profile of 10 L mass culture system. open circle: without ROCK inhibitor (*n* = 1), closed circle: with ROCK inhibitor (*n* = 3). (B) Representative images of hiPSC aggregates without ROCK inhibitor. Scale bars, 200 µm.


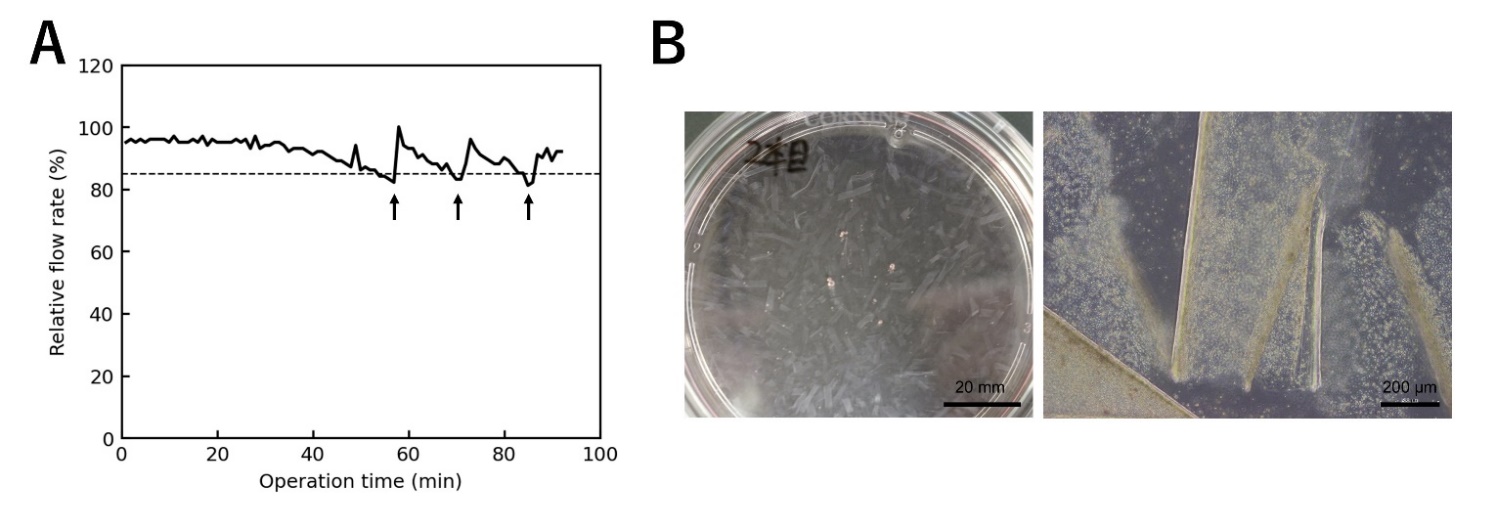


**Supplementary Figure 6.** Effect of backwash operation in the medium exchange process. (A) Relationship between operation time and relative flow rate. Relative flow rate is given in percent values against the set value of flow rate. Arrow indicates the backwash operation. (B) Representative images of debris in the backwash waste.
